# Increasing preparedness for caregiving and death in family caregivers of patients with severe illness who are cared for at home – study protocol for a web-based intervention

**DOI:** 10.1186/s12904-020-0530-6

**Published:** 2020-03-17

**Authors:** Anette Alvariza, Louise Häger-Tibell, Maja Holm, Gunnar Steineck, Ulrika Kreicbergs

**Affiliations:** 1 The Department of Health Care Science/Palliative Research Centre, Ersta Sköndal Bräcke University College, Stigbergsgatan 30, 100 61 Stockholm, Sweden; 2 Capio Palliative Care Unit, Dalen Hospital, Åstorpsringen 6, 121 31 Enskededalen, Sweden; 3 Tema Cancer, BES: Breast-endocrine tumors and sarcoma, Karolinska University Hospital, Eugeniavägen 3, 171 76 Solna, Sweden; 4 Department of Nursing Science, Sophiahemmet University, Valhallavägen 91, 114 28 Stockholm, Sweden; 5 Division of Clinical Cancer Epidemiology, Department of Oncology, Institute of Clinical sciences, Sahlgrenska Academy at the University of Gothenburg, Blå Stråket 2 SU/Jubileumskliniken, 413 45 Gothenburg, Sweden; 6 Department of Oncology and Pathology, Division of Clinical Cancer Epidemiology, Karolinska Institutet, Solnavägen 1, 171 77 Solna, Sweden; 7 Department of Women’s and Children’s Health, Karolinska Institutet, Solnavägen 1, 171 77 Solna, Sweden

**Keywords:** Web-based intervention, Palliative care, Family caregivers, Severe illness, Preparedness

## Abstract

**Background:**

Family caregivers of patients with severe illness and in need for a palliative care approach, face numerous challenges and report having insufficient preparedness for the caregiver role as well as a need for information and psychosocial support. Preparing to care for a severely ill family members also means becoming aware of death. Feelings of being prepared are associated with positive aspects and regarded protective against negative health consequences.

**Methods:**

The study adheres to the SPIRIT-guidelines (Supplementary 1), uses a pre-post design and include a web-based intervention. Inclusion criteria are; being a family caregiver of a patient with severe illness and in need of a palliative care approach. The intervention which aims to increase preparedness for caregiving and death is grounded in theory, research and clinical experience. The topics cover: medical issues, symptoms and symptom relief; communication within the couple, how to spend the time before death, being a caregiver, planning for the moment of death and; considerations of the future. The intervention is presented through videos and informative texts. The website also holds an online peer-support discussion forum. Study aims are to: evaluate feasibility in terms of framework, content, usage and partners’ experiences; explore how the use of the website, influences family caregivers’ preparedness for caregiving and death; explore how the use of the website influences family caregivers’ knowledge about medical issues, their communication with the patient and their considerations of the future; and to investigate how the family caregivers’ preparedness for caregiving and death influences their physical and psychological health and quality of life 1 year after the patient’s death. Data will be collected through qualitative interviews and a study-specific questionnaire at four time-points.

**Discussion:**

This project will provide information about whether support via a website has the potential to increase preparedness for caregiving and death and thereby decrease negative health consequences for family caregivers of patients affected by severe illness. It will provide new knowledge about intervention development, delivery, and evaluation in a palliative care context. Identification of factors before death and their association with family caregivers’ preparedness and long-term health may change future clinical work.

**Trial registration:**

The study is registered at ClinicalTrials.gov: NCT03676283.

## Background

Although there are great variations, family caregivers of patients with severe illness often face numerous tasks involving the practical and medical caregiving for the patient while also providing emotional, social and existential support as well as maintaining household chores [1]. They may or may not recognize themselves as caregivers; nevertheless, the scientific literature defines a family caregiver as any relative, friend, or family caregiver who has a significant relationship with, and provides various forms of assistance to, an ill person. Thus, the term ‘caregiving’ covers a broad perspective [2]. Family caregivers often report being insufficiently prepared for the caregiver role as well as a consequent need for information and psychosocial support [3, 4]. Preparedness for caregiving refers to how ready the family caregivers themselves perceive they are for the tasks related to the caregiving role, such as providing physical care, emotional support and dealing with the stress of caregiving. Thus, preparedness for caregiving has both a practical and emotional component; knowing what to do, but also coping with emotions and stress [5]. Feelings of being prepared are associated with several positive aspects, such as less burden and anxiety, and can be regarded as a protective factor against negative consequences. Preparedness may also promote stronger feelings of reward and hope as well as better health [6, 7]. Increased feelings of preparedness for family caregivers have been suggested to possibly affect the patient in a favorable way [8].

In palliative care, family caregivers also need to prepare for the patient’s death [3]. Preparedness for death refers to being aware that an impending death may occur at any moment. Through interviews, it has been found that preparing to care for a severely ill family member also means becoming aware of death [9–11]. Awareness of death can be frightening and can result in a very tense situation [11], however, it can also be helpful; contributing to a sense of having control over the situation [9]. Results from a nation-wide study of widowers showed that a high level of preparedness promoted their long-term well-being [12]. In one study of a bereaved population, 25% reported that health-care professionals could have done more to prepare the bereaved for the death of their loved one. The bereaved believed that the physicians’ lack of communication concerning the prognosis and impending death hindered them from being able to prepare [13]. Results from other studies have indicated that end-of-life discussions, preparedness for death, encouragement of advanced care planning and reducing family caregivers’ distress before the loss, may also improve adjustment after the loss [14, 15]. Most people seem to adjust to the caregiver role [16, 17] and to the loss of a close person and recover from the emotional strain. However, some suffer beyond what can be viewed as normal grief, i.e., they experience a long-lasting decline in their mental and physical health [18–21] and are even at an increased risk of death [20, 22]. Interventions with various designs have had significant positive effects on family caregivers’ feelings of preparedness for caregiving [23–25]. Intervening before the death of the patient, providing more information and support, and thereby increasing preparedness, may also lessen the burden of bereavement.

Most interventions were earlier delivered either face-to-face or in written documents. Now the internet is a natural platform for distributing information, with its unique advantage of easy accessibility and the potential to diminishing geographic and time barriers. The use of web-based interventions to support family caregivers has increased lately. A total of 12 systematic literature reviews [26–37] report on various web-based interventions, half of them focusing on family caregivers of patients diagnosed with dementia, and the others focusing on caregivers of patients affected by cancer or chronic conditions. The reviews all conclude that content, structure and duration differ markedly between the reported interventions, which included various designs; online support groups, informational websites, websites combined with telephone support or email support, and websites combined with exchange with other caregivers online. Altogether, the reviews report a range of improved family caregiver outcomes, such as increased knowledge, self-efficacy, self-esteem, coping skills, wellbeing and reduced anxiety, depression, stress, strain, and burden [26]. Overall, the reviews conclude that web-based family caregiver interventions can be beneficial in offering information and support, but more research is needed.

The strained situation for family caregivers of patients affected by severe illness and in need of a palliative care approach is by now known, and the positive influence of feeling prepared has now been proven [26]. Several web-based interventions to support family caregivers have been developed and are reported to have positive outcomes and improvements for family caregivers. However, there is still a need to examine whether a web-based intervention can be used in the context of palliative care to promote family caregivers’ preparedness for caregiving and death. Thus, the aim of this paper is to describe a study protocol which addresses this subject.

## Methods

### Design

The study adheres to the SPIRIT guidelines (Supplementary 1) and uses a pre-post design to evaluate an intervention that is delivered and presented on the website narstaende.se. The intervention is a complex intervention, as defined by the Medical Research Council guidance [38, 39], because it comprises a number of interacting components, a number of behaviors required by those receiving the intervention, and a number of outcomes and flexibility in the use of the intervention. The study follows the steps that are significant in considering a complex intervention, which are: development based on existing evidence and theory, assessing feasibility, assessing effectiveness, understanding processes, and measuring outcomes. The study is registered at ClinicalTrials.gov NCT03676283.

### The intervention

#### Intervention content and design

The overall aim of the intervention is to increase preparedness for caregiving and death in family caregivers of patients affected by severe illness and to decrease the risks of negative health consequences. The intervention content rests on evidence and previous research, as well as clinical experience, and addresses topics shown to be associated with preparedness. The topics cover: a) medical issues, including symptoms and symptom relief; b) communication within the couple, how to spend the time before death, being a family caregiver or caregiver, planning for the moment of death; and c) considerations of the family caregiver’s future, including psychological issues, logistical issues, finances, and the care of children (Table 1). The intervention is presented in the Swedish language at the website närståede.se through videos and informative texts. The videos show conversations, addressing intervention topics between family caregivers (played by actors) and different health-care professionals (authentic). The informative texts complement, deepen and broaden the topics raised in the videos. As a part of the intervention, there is also an online peer-support discussion forum at the website, which can be used by family caregivers to communicate with others in a similar situation. The website narstaende.se, solely hosts the intervention and is co-designed between health-care researchers, clinical health-care professionals, information systems researchers, a digital communication strategist and IT-consultants. The website will be accessible from mobile phones, tablets and computers. All interaction with the site, and the related information and user credentials, is encrypted and secured according to the GDPR directive (EU 2016/679).

**Table 1:** Topics addressed in films and texts

To think through whether she or he spends as much time with the patient as she or he wants.	
To think through the dual role that might evolve as being a family member and a caregiver. To think through whether she or he participates in the patient’s care according to her or his own wishes.	
To communicate with the patient about the disease’s physical aspects in a structured way. To have knowledge of common symptoms that may occur, such as pain, constipation and others, and how to deal with them.	
To communicate with the patient about funeral-related issues.	
To communicate with the patient about financial questions and writing a will.	
To communicate, if applicable, with the patient about their children’s well-being and future.	
To communicate with the patient about her or his psychological symptoms and feelings relating to the impending death.	
To communicate, if appropriate, with the patient about emotional issues from the past, such as unfinished business, coming to closure, forgiveness and giving thanks.	
To imagine herself or himself as bereaved 6 months after the patient’s death, and considering the psychological and practical implications.	
To imagine, cognitively and emotionally, the patient’s death.	

#### Theoretical framework

In addition to the body of empirical research about preparedness for caregiving and death, Andershed and Ternestedt’s theoretical framework [40] was used in guiding the development and design of the intervention. The framework derives from qualitative data and focuses on the involvement and principal needs of family caregivers in palliative care and is used here to enhance the understanding of the caregiver situation. The framework is clearly written on an abstract level but is relatively easy to absorb and could therefore serve as a guide for practice. The framework illustrates three components of principal needs; these are knowing, being and doing. These components are closely related to the concept of preparedness. Knowing represents family caregivers’ need for, and active seeking of, knowledge, which is a prerequisite for being and doing. Family caregivers try to increase their understanding of the patient’s condition by actively seeking knowledge of, for example, the patient’s symptoms, diagnosis, and prognosis. Being concerns emotional aspects, such as spending time with the patient and sharing his or her world. Doing has a more practical aspect and involves the family caregiver doing things for the patient, such as helping the patient with hygiene or medication, or keeping in contact with friends and family [41]. The theoretical framework further strengthens the content and the choice of topics in the intervention, which is tailored to meet the family caregivers’ need for knowing, being and doing.

### Aims

The overall aim of the research is to examine the use of and potential influences of a web-based intervention targeting family caregivers of patients affected by severe illness who are cared for at home. To reach a deeper understanding for such an approach, the following four specific aims will be addressed:

- Specific aim I: To evaluate feasibility in terms of framework, content, usage and family caregivers’ experiences of a web-based psycho-educational intervention. *Family caregivers will be interviewed and statistics concerning their use of the website will be used.*
- Specific aim II: To explore how the use of a website influences family caregivers’ degree of preparedness for caregiving and death. *Preparedness for caregiving will be measured at baseline and 4 weeks later. Preparedness for death and associations with preparedness for caregiving will be measured 8 weeks after the patient’s death. Interviews will also be used for data collection.*
- Specific aim III: To explore how the use of a website influences family caregivers’ knowledge about medical issues, their communication with the patient, and their considerations of the future. *Measurements will be performed at baseline and 4 weeks later. Interviews will also be used for data collection.*
- Specific aim IV: To investigate how family caregivers’ degree of preparedness for caregiving and death influences their physical and psychological health and quality of life 1 year after the patient’s death. *Information collected as described for the specific aims II and III will be related to physical and psychological health, measured 1 year after the patient’s death.*

### Inclusion criteria for patients and family caregivers

This study focuses on family caregivers of patients affected by severe illness who are enrolled in specialized home care. The inclusion criteria are; being a family caregiver, over 18 years old, of a patient affected by severe illness and in need of a palliative care approach (“It is probably a matter of months, not years”). Severe illness is here understood as any acute or chronic illness and/or condition that causes significant impairment, and may lead to long-term impairment, disability and/or death [42]. Equally important is that the patient must have a cognitive function sufficient for communication. The patient should have been informed that his or her illness cannot be cured. Family caregivers need to understand and speak Swedish. To obtain enough data after attrition, we plan to recruit 200 family caregivers.

### Recruitment

**Fig. 1:**
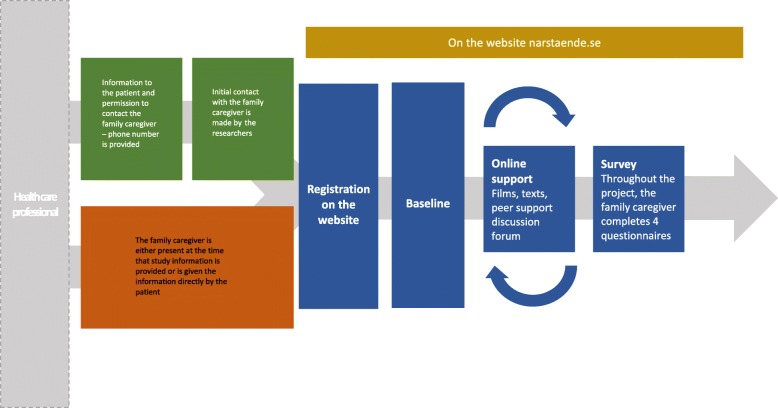
Flow chart of the research process

Altogether, directors of departments covering 30 specialist palliative home care units have given written informed consent for recruitment of family caregivers. The home care units are staffed with multi-disciplinary teams of health-care professionals, including nurses, physicians, physiotherapists, occupational therapists, dieticians and social workers delivering 24-h care in private homes. Health-care professionals employed in the settings will recruit family caregivers, according to the inclusion criteria, until the recruitment target has been reached, which is expected to be within a period of approximately 1 year and a half. Initially, the patient will be approached by a health-care professional who will provide them with written information about the study. The health-care professional will also ask the patient whether the researchers have their permission to contact his or her family caregiver/s for information about the study. The patient can thus nominate one or more family caregivers to the study. If a family caregiver is present at the time of recruitment, he or she will receive the information by the same time as the patient. If the patient agrees that his or her family caregiver/s can be contacted, two different scenarios can take place: 1) the health-care professional is given a telephone number for the family caregiver/s and registers the number in an administrative database, whereupon a research assistant calls the family caregiver/s to provide study information and a request to participate; or 2) the patient informs the family caregiver/s or the family caregiver/s has been present at the time for recruitment and pays a visit to the website on his or her own initiative. After agreeing to consider participation, the family caregiver/s registers at narstaende.se, where they have access to text informing them about the study and where they can give their consent to participate. If they agree to participate, the family caregivers will be asked to complete a baseline questionnaire electronically (Fig. 1).

All participants will receive a telephone call from one of the researchers to help them access the web page. All participants will also be able to call for support in the use of the website. If needed, the researcher will provide instructions for navigating the website to help the participants to access the videos, text, and the peer-support communication forum. Being familiar with the content of each text and video, the researcher can guide family caregivers to specific content when a specific issue is raised.

### Outcomes

The study focuses on preparedness for caregiving and preparedness for death as these concepts have earlier been shown to be clearly and closely related to each other in the context of palliative cancer care. The secondary outcomes are: changes in family caregivers’ knowledge about medical issues, their communication with the patient, and their considerations of the future. These concepts are related to the topics that the intervention addresses. Other secondary outcomes will be the physical and psychological health and quality of life for the family caregiver 1 year after the patient’s death. Outcomes will also relate to the feasibility of the website’s framework, content, uses and family caregivers’ experiences of using the website.

Information about which participants have logged on to narstaende.se, which videos have been watched and how many times a specific video was watched can be gathered electronically. Information on how long the participant stays on certain pages can also be obtained. These factors can thus be studied in relation to preparedness for caregiving as well as preparedness for death.

### Data collection and measures

Data will be collected with the use of qualitative interviews and a study-specific questionnaire that has been developed based on earlier studies and in face-to-face validation [43–45]. The questionnaire includes:

- background variables such as age, gender, and education,
- the Preparedness for Caregiving Scale, which measures perceived readiness for various domains of caregiving [5]. It has been translated into Swedish and has demonstrated good validity and reliability for caregivers in palliative care [43, 46]. It consists of eight items answered on a 5-point Likert-type response scale, ranging from ‘not at all prepared’ (0) to ‘very well prepared’ (4), with a total score ranging from 0 to 32.
- single items covering the intervention topics: a) medical issues including symptoms and symptom relief; b) communication within the couple, how to spend the time before death, being family member and caregiver, planning for the moment of death; and c) considerations of the family caregiver’s future, including psychological issues, logistical issues, finances, and the care of children.
- single items about psychological health and quality of life

Feasibility will be evaluated through semi-structured interviews (aim I). Preparedness for caregiving will be measured at baseline and 4 weeks later (aim II). Preparedness for death and associations with preparedness for caregiving will be measured 8 weeks after the patient’s death (aim II). Intervention topics will be measured at baseline and 4 weeks later (aim III). Family caregivers’ physical and psychological health and quality of life will be measured 1 year later (aim IV).

### Data analyses

#### Analysis of interviews

For aim I, data from the semi-structured interviews with participants will be analyzed through qualitative content analysis [47].

#### Statistical analysis

In the analysis of preparedness for caregiving (aim II), we will address the statistical null hypothesis by analyzing mean differences between two dependent means (pre- and post-intervention). Cut-off values for low, moderate and high preparedness have been established through earlier studies [24] and will be applied to the results. Preparedness for death (aim II) will be examined using descriptive statistics, and associations with preparedness for caregiving will be investigated with the use of linear regression analysis. Guided by the intention-to-intervene (intervention to treat) principle, we will include all family caregivers for whom we have information on the respective outcome. For aims III and IV we will proceed in a similar manner.

#### Statistical power

Statistical power was calculated based on the preparedness for caregiving scale [5, 46]. As a model, we used a previous intervention study [24], where a statistically significant (alpha = 0.05) improvement in preparedness for caregiving was observed between two dependent means (*t*-tests) with an effect size of 0.27. In order to obtain statistical power of 80%, we would need 109 participants. However, with regard to the number of attritions in the previous intervention study, we decided to raise the required sample to 200 participants.

#### Validity

This trial has a pre- and post-intervention design. Because there is little previous research on this subject, this trial could be considered as an important first step to explore whether web-based interventions can be used to effectively support family caregivers in palliative care. The website and its content will probably improve during the project time, not least through the content that will accumulate on the forum used by the participating family caregivers themselves. This possible effect-modifying factor will be studied by using the variable “calendar time since trial start”.

### Ethical considerations

The family caregivers of patients who are at risk of dying within months are in a particularly vulnerable situation. We have considered that watching the videos and answering the questionnaires could be associated with strong emotions and, for this reason, written and oral study information will emphasize the voluntary nature of participation and the right to withdraw from the study without any explanation. When entering, analysing and presenting the results, data will be treated according to the principle of confidentiality, and the identity of participants will be protected. The participants’ names will be replaced with a code and the list of coded names will be kept in locked storage, accessible only to the researchers of the study. A regional ethical committee has approved the study (no: 2018/1893–31).

## Discussion

This novel project will add to the increasing body of knowledge on web-based interventions and will provide information about whether support offered via a website has the potential to increase preparedness and decrease the negative health consequences for family caregivers of patients affected by severe illness and in need for a palliative care approach. Through scientific publications, presentations at clinical research congresses and lectures at health-professional education programs, the project will contribute to new knowledge about intervention development, delivery, and evaluation in a palliative care context. The identification of factors before death and their association with family caregivers’ preparedness and long-term health may change future clinical work. Further, rapid demographic changes and societal developments make this project important. Aging populations, combined with expanded home care, places a great weight on family caregivers and will continue to make their efforts as informal caregivers crucial. Web-based interventions make it possible to support many family caregivers with fewer resources. Some family caregivers may find it difficult to attend face-to-face due to their heavy care burden; for them, a web-based intervention might seem more appealing and not all caregivers are comfortable with attending support groups in person. The project might narrow a gap also for health-care professionals, as it may be easier for them to reach this vulnerable group on the web. Today, the internet is a natural part of many people’s lives and it is common to seek support regarding health-care issues online. While older people are less likely to use the internet than the young, internet use is constantly increasing across all age groups.

A psycho-educational intervention using similar content and topics as the web-based intervention presented in this study protocol was delivered to family caregivers and other family members during ongoing palliative care and was shown to be effective, both in promoting preparedness for caregiving and preparedness for death [24, 25]. In addition, the program was experienced as being valuable, both by family members and health-care professionals. No negative effects were reported as a result of delivering the intervention.

Barriers to intervention effectiveness may be selection bias among participants, a bias that may be reduced by using the web rather than a face-to-face approach. When an opportunity is given to take part in the intervention via a website, it is reasonable to assume that family caregivers who are under more stress and those who have greater needs will be able to participate and thereby receive support aimed at increasing preparedness and decreasing risks of negative health consequences.

## Supplementary information

**Additional file 1:** SPIRIT 2013 Checklist: Recommended items to address in a clinical trial protocol and related documents*.

## Data Availability

The data used and/or analysed during the current study will be made available from the corresponding author upon reasonable request.

## Abbreviations

EU: European Union
GDPR: General Data Protection Regulation
IT: Information technology
